# Reduced finger tapping speed in patients with schizophrenia and psychomotor slowing: an exploratory fMRI study

**DOI:** 10.3389/fpsyt.2025.1539112

**Published:** 2025-04-28

**Authors:** Florian Wüthrich, Marc O. Zindel, Niluja Nadesalingam, Melanie G. Nuoffer, Alexandra Kyrou, Jessica A. Bernard, Stewart A. Shankman, Vijay A. Mittal, Stephanie Lefebvre, Sebastian Walther

**Affiliations:** ^1^ Translational Research Center, University Hospital of Psychiatry and Psychotherapy, University of Bern, Bern, Switzerland; ^2^ Translational Imaging Center (TIC), Swiss Institute for Translational and Entrepreneurial Medicine, Bern, Switzerland; ^3^ University Hospital of Old Age Psychiatry and Psychotherapy, University of Bern, Bern, Switzerland; ^4^ Graduate School for Health Sciences, University of Bern, Bern, Switzerland; ^5^ Department of Psychological and Brain Sciences, Texas A&M University, College, Station, TX, United States; ^6^ Texas A&M Institute for Neuroscience, Texas A&M University, College, Station, TX, United States; ^7^ Institute for Innovations in Developmental Sciences, Northwestern University, Evanston, IL, United States; ^8^ Institute for Innovations in Developmental Sciences, Northwestern University, Chicago, IL, United States; ^9^ Medical Social Sciences, Northwestern University, Chicago, IL, United States; ^10^ Department of Psychiatry and Behavioral Sciences, Northwestern University, Chicago, IL, United States; ^11^ Department of Psychology, Northwestern University, Chicago, IL, United States; ^12^ Institute for Policy Research, Northwestern University, Chicago, IL, United States; ^13^ Department of Psychiatry, Psychosomatics and Psychotherapy, Center of Mental Health, University Hospital of Würzburg, Würzburg, Germany

**Keywords:** schizophrenia, psychosis, fMRI, task-fMRI, finger-tapping, psychomotor slowing

## Abstract

**Introduction:**

Motor symptoms are frequent in patients with schizophrenia and have multiple presentations, one of which is psychomotor slowing. Understanding the neural basis of psychomotor slowing may help improve future therapies in schizophrenia. Here, we performed task-fMRI using a finger-tapping task in slowed patients.

**Methods:**

The study included 36 patients with schizophrenia and psychomotor slowing (Salpêtrière-Retardation-Rating-Scale-Score (SRRS) >15), 11 non-slowed patients with schizophrenia, and 33 healthy controls who successfully performed a motor task during fMRI, with four conditions: paced and fast thumb-index finger tapping and thumb alternating finger opposition. The performance was videotaped and taps were counted. We compared task-related neural substrates between groups, task complexity and movement onset.

**Results:**

Slowed patients with schizophrenia showed significantly lower tapping speed than controls in both unpaced conditions (Δ=-.80 (CI=-1.46; -.14)taps/s, p=.019; Δ=-.80 (CI=-1.32; -.28)taps/s, p=.003) while non-slowed patients had a tapping speed between the other two groups.

**Discussion:**

In both task complexity and movement onset factor levels, all the groups activated sensorimotor areas. Slowed patients had no regulation of the task-dependent cerebellar involvement while showing insufficient deactivation of the SPL, pointing to altered recruitment of neural resources in response to motor demands in schizophrenia especially when associated with psychomotor slowing.

## Introduction

Motor abnormalities are a frequent symptom of schizophrenia. One of these abnormalities is psychomotor slowing, which is characterized by slowed mental and movement processes such as reduced concentration, slowed processing speed, gait, or movements, decreased overall activity, spontaneous movements, or reduced facial expressions (1–3). Psychomotor slowing is associated with poor social, physical and mental outcome in schizophrenia: Patients with psychomotor slowing have higher disability, more sedentary behaviour and cardio-metabolic risk, feel worse and have a lower quality of life (4–7). Moreover, we have recently shown that psychomotor slowing in schizophrenia is associated with poor functioning, more negative symptoms, and other motor abnormalities (8–10). Specific treatments for psychomotor slowing in schizophrenia are emerging, such as brain stimulation techniques (11–13). However, knowledge about the neural correlates of psychomotor slowing in schizophrenia is required to refine current and develop novel treatment approaches (11).

Motor abnormalities are associated with functional and structural alterations in the cerebral motor system (14). While highly interconnected, three main circuits governing different aspects of motor behaviour can be distinguished: the basal ganglia circuit controlling the excitation/inhibition balance, the cerebello-thalamic circuit involved in sensorimotor integration and online updating of movement, and the cortical motor circuit responsible for psychomotor organization (15, 16). Indeed, aberrant motor behaviour in schizophrenia is associated with grey matter volume changes in cortical and subcortical structures of the motor system (17–21). In a recent study, psychomotor slowing was associated with cortical thinning of the primary motor cortex, but this finding was not reproducible in two independent cohorts (22). Measures of psychomotor slowing are also associated with reduced white matter integrity and altered structure in various tracts, such as the cingulum, corpus callosum, longitudinal fasciculus, or the internal and external capsule (23–27). With regard to tasks, lower activation in the primary and premotor cortices, dorsolateral prefrontal cortex and in basal ganglia are associated with slower performance (28, 29, 30). Moreover, aberrant functional connectivity between cortical and subcortical regions was linked to psychomotor slowing in schizophrenia (26, 31, 32). Psychomotor slowing is associated with alterations of primary motor cortex physiology. Recently, we have shown lower amplitudes of motor evoked potentials and diminished cortical inhibition in patients with schizophrenia and psychomotor slowing compared with controls. Moreover, primary motor cortex physiology was differentially associated with measures of structural and functional connectivity in patients with and without psychomotor slowing (33).

Finger-tapping is a relatively simple motor task that can be executed in the confined space of an MR scanner. Studies examining the neural responses to finger-tapping in schizophrenia found lower activation in pre- and postcentral gyrus, supplementary motor area, and cerebellum in patients compared with controls and treatment-naïve patients showed overactivation of subcortical structures (34–38). However, these studies only compared patients with schizophrenia and healthy controls. Since patients with schizophrenia and psychomotor slowing show different associations of physiology and imaging measures than patients without slowing, the response to tasks may differ between these patient groups as well. To examine the specific effect of psychomotor slowing on neural responses to finger-tapping, we compared schizophrenia patients with and without psychomotor slowing, as well as healthy controls.

We hypothesized that patients with psychomotor slowing would exhibit a lower tapping frequency than the other groups during the unpaced conditions. We expected hypoactivations in regions of the cortical motor circuit, especially the primary motor cortex. Recently, the cognitive dysmetria hypothesis of schizophrenia has regained interest, and altered cerebellar structure or connectivity as well as associations with motor function have been shown (32, 39, 40). Therefore, we also expected altered activation of the cerebello-thalamic circuit.

## Materials and methods

### Participants

Participants in this study were recruited for the OCoPS-P trial (12, 33) (ClinicalTrials.gov identifier: NCT03921450). From the 168 participants of the OcoPS-P baseline (99 slowed patients with schizophrenia (PS), 27 non-slowed patients (non-PS), and 42 healthy controls (HC)), only 80 participants (36 PS, 11 non-PS, and 33 HC) were included in the analyses (demographics: **Table 1**; participant flow: **Supplementary Figure S1**). The main criteria for exclusion were the refusal by patients to perform the task (N=10) and the patient’s inability to perform the task, i.e: no correct execution of at least one condition either during the training period outside the scanner (N= 41) or during the MRI acquisition (N=13), other reasons included technical issues (N=3), language issues (N=4), left-handedness as confirmed by the Edinburgh Handedness Inventory (41) (N=4), and image quality issues (excessive motion defined as mean framewise displacement >.5 mm, total translation or rotation in any axis > 3 mm; benign incidental tumour; excessive signal extinction, N=13).

**Table 1:** Study population characteristics.

	PS (n=36) Mean (SD)	non-PS (n=11) Mean (SD)	HC (n=33) Mean (SD)	Statistics	
Age (years)	31.4 (9.2)	32.4 (12.2)	36.6 (12.6)	F_(2,77)_=2.0, p=.73	
Sex nf (%)	17 (51.5)	7 (63.6)	18 (50.0)	χ^2^=.65, p=.72	
Education (years)	13.4 (1.6)	12.4 (.5)	16.3 (3.3)	F_(2,77)_=17.6, p<.001	HC>non-PS: p<.001 HC>PS: p<.001
SRRS	24.3 (5.9)	8.2 (2.2)	.4 (.8)	F_(2,77)_=306.1, p<.001	non-PS>HC: p<.001 PS>HC: p<.001 PS>non-PS: p<.001
Medication (OLZ_eq_)	16.1 (12.6)	17.8 (12.8)	–	T=-.41, p=.69	
Medication (diazepam_eq_)	1.6 (5.9)	4.8 (6.5)	–	T=1.49, p=0.12	
PANSS total	79.8 (17.4)	59.0 (12.6)	–	T=4.4, p<.001	
BFCRS	4.9 (3.0)	.7 (.8)	–	T=7.6. p<.001	
UPDRS	19.9 (10.2)	10.8 (6.3)	–	T=3.6, p=.001	
SOFAS	45.3 (13.9)	49.5 (13.3)	91.4 (5.4)	F_(2,77)_=178.5 P<.001	HC>non-PS: p<.001 HC>PS: p<.001 non-PS>PS: p=.14
Coin rotation	11.9 (3.6)	12.3 (3.4)	14.4 (3.6)	F_(2,77)_=4.6, p=.01	HC>non-PS: p=.21 HC>PS: p=.01 non-PS>PS: p=.93
Tapping TIF*_unpaced_* (taps/s)	3.03 (1.30)	3.32 (1.05)	3.73 (1.14)	F_(2,77)_=3.0, p=.057	HC>non-PS: p>.05 HC>PS: p>0.05 non-PS>PS: p>.05
Tapping TAF*_unpaced_* (taps/s)	2.16 (.83)	2.63 (.84)	3.00 (.90)	F_(2,77)_=8.1, p<.001	HC>non-PS: p>.05 HC>PS: p<.001 non-PS>PS: p>.05

PS, patients with slowing; non-PS, patients without slowing; HC, healthy controls; SRRS, Salpêtrière retardation rating scale; OLZ_eq_, olanzapine equivalents; diazepam_eq_, diazepam equivalents; PANSS, positive and negative syndrome scale; BFCRS, Bush-Francis catatonia rating scale; UPDRS, unified Parkinson’s disease rating scale; SOFAS, social and occupational functioning scale; TIF, thumb-index finger tapping; TAF, thumb-alternating finger opposition.

Patients were recruited from the in- and out-patient departments of the University Hospital of Psychiatry and Psychotherapy in Bern, Switzerland. Healthy controls were recruited from the general population. Patients were eligible for inclusion if they had been diagnosed with schizophrenia spectrum disorders according to DSM-5. Exclusion criteria for both groups included age lower than 18 or higher than 65 years, active substance dependence other than nicotine, neurological or musculoskeletal disorders affecting motor abilities, epilepsy, history of severe brain injury with consecutive loss of consciousness for several minutes, and MR-contraindications.

All participants provided written informed consent. The study protocols adhered to the Declaration of Helsinki and were approved by the local ethics committee (KEK-BE 2018-02164).

### Clinical assessments

We confirmed diagnosis in patients using the Structured Clinical interview for DSM-5 (SCID) and clinical case files. We considered psychomotor slowing to be present if patients had a score >15 on the Salpêtrière-Retardation-Rating-Scale (SRRS) (42). The SRRS is a commonly used tool for assessing psychomotor slowing in depression and psychosis. Although no definitive cut-off has been established for this scale, the literature reports cut-offs ranging from 10 to 20. In the last decade, our team has conducted several studies using the cut-off of 15 points which would suggest an impairment on several motor and cognition-related items. This classification resulted in categorizations that align closely with those derived from behavioral assessments such as actigraphy. (8, 9, 12, 33, 43) Symptom severity was evaluated with the Positive And Negative Syndrome Scale (PANSS) (44), catatonia with the Bush-Francis Catatonia Rating Scale (BFCRS) (45), parkinsonism with the Unified Parkinson’s Disease Rating Scale part III (UDRS) (45), functioning with the Social Occupational Functioning Scale (SOFAS) (46), and manual dexterity was assessed with the coin rotation task (CR) (12, 47). We converted antipsychotic medication dosage to olanzapine equivalents (OLZ_eq_) and benzodiazepines medication dosage to diazepam equivalent (diazepam_eq_) according to (48).

### Image acquisition

We performed imaging at the Translational Imaging Center Bern of Sitem-insel Bern on a 3T Magnetom Prisma scanner (Siemens Healthcare, Erlangen, Germany). First, we acquired structural T1-weighted images (MPRAGE, 176 slices, FOV 240 x 256 mm, voxel size 1x1x1mm, TR=5000ms, TE=2.98ms, flip angles=4°/5°) and then task-based fMRI (multiband accelerated echo-planar BOLD images, 660 volumes, covering 11 minutes, 72 slices, FOV 230x230mm, voxel size 2.5x2.5x2.5mm, TR=1000ms, TE=37ms, flip angle=30°).

### fMRI task

**Figure 1:**
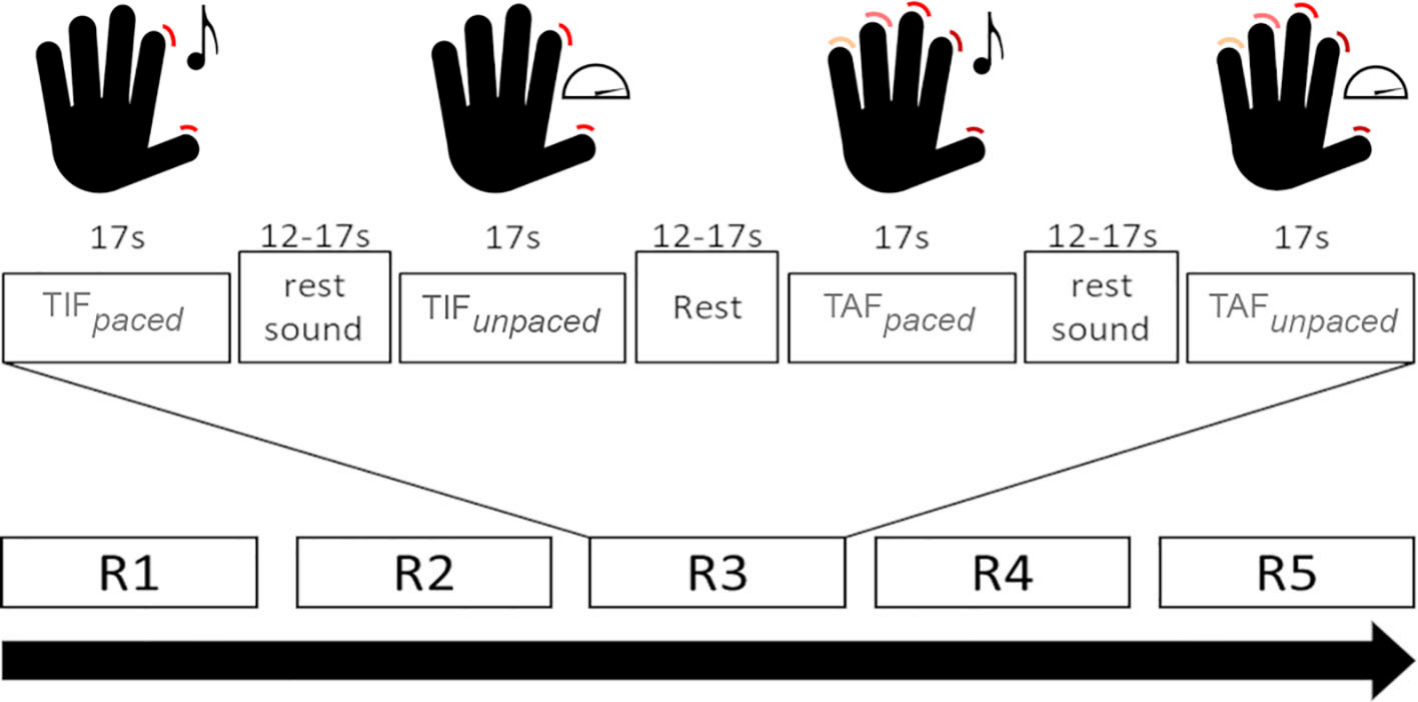
Schematic depiction of the task conditions. TIF*_paced:_* Sound-paced thumb-index finger tapping; TIF*_unpaced_*: unpaced thumb-index finger tapping; TAF*_paced_*: Sound-paced thumb-alternating finger opposition; TAF*_unpaced_*: unpaced thumb-alternating finger opposition; Rx: run number x. Adapted Figure from Wüthrich et al. (49) under CC license.

Details of the task and its test-retest reliability in healthy participants have been reported elsewhere (49). In short, participants performed a finger-tapping task with their dominant (right) hand, including four active and two rest conditions. This was a block design with five runs. Active conditions had a duration of 17 seconds, rest conditions one of 12 – 17 seconds. Active conditions included: 1. Sound-paced thumb-index finger tapping at 0.5 Hz (TIF*_paced_*); 2. Unpaced, fast thumb index finger tapping (TIF*_unpaced_*); 3. Sound-paced thumb-alternating finger opposition at 0.5 Hz (TAF*_paced_*); 4. Unpaced, fast thumb-alternating finger opposition (TAF*_unpaced_*). Each paced condition was followed by a rest condition during which the sound continued but the instruction was to do nothing. The second rest condition followed TIF*_unpaced_* and did not include any stimuli. The order of conditions was fixed and the same in all runs and participants (**Figure 1**). We videotaped the task execution. An investigator confirmed the correct execution and counted the number of taps based on these recordings. Participants with at least one correct instance of every condition were included in further analyses.

### Preprocessing

We applied the identical preprocessing pipeline as described previously (49) using SPM12 (Revision 7771, Welcome Trust, London, U.K., https://www.fil.ion.ucl.ac.uk/spm/) and MATLAB (R2020b, MathWorks, Natick, USA). We performed segmentation of structural images with CAT12 (http://www.neuro.uni-jena.de/cat/), normalization and smoothing of structural images with a 5mm FWHM kernel using Dartel (50). Then we realigned functional images, coregistered them to the corresponding structural image, and applied the same normalization fields and smoothing parameters as for the structural images. We censored blocks with excessive motion limited to one task condition (framewise displacement > 2mm calculated by the Power-method (51)). Participants with excessive residual movement (mean framewise displacement >.5mm, total translation or rotation in any axis > 3mm) were excluded from further analysis.

### Statistical analyses

Statistical analyses were carried out in R (The R Foundation, V. 4.2.2) and SPM12. We compared demographics, clinical characteristics, and tapping performance in the unpaced conditions between PS, non-PS, and HC using Chi-square tests and one-way ANOVAs where appropriate. *Post-hoc* tests were carried out using Tukey honest significant difference. To assess neural response to the task conditions, we first performed a first-level analysis in SPM12 by building a general linear model with one regressor for each active and both rest conditions. We included the six movement parameters (x-, y-, z-translations and rotations) as covariates to account for residual motion. Each active condition was contrasted with the corresponding rest condition.

To compare task-related neural substrates between groups, we performed a whole-brain 3-ways full factorial design (3x2x2) with three factors: group (three levels: HC, non-PS, and PS), task complexity (two levels: TIF (All conditions using TIF) and TAF (All conditions using TAF)), and movement onset (two levels: paced (All paced conditions) or unpaced (All unpaced conditions)), with age and sex as covariates. Additionally, we replicated this design including only the patient groups (2x2x2), with three factors: group (two levels: non-PS and PS), task complexity (two levels: TIF (All conditions using TIF) and TAF (All conditions using TAF)), and movement onset (two levels: paced (All paced conditions) or unpaced (All unpaced conditions)), including age, sex, general disease severity assessed by PANSS total, and current antipsychotic and benzodiazepine medication dosages (olanzapine and diazepam equivalents) as covariates. Then, to account for potential effects between task conditions not captured by the factors complexity and movement onset, we performed a 2-ways full factorial design, two factors: group (three levels: HC, non-PS, and PS and conditions (four levels: TIF*_paced_*, TAF*_paced_*, TIF*_unpaced_*, TAF*_unpaced_*). The three statistical models are summarized in **Table 2**.

**Table 2:** Statistical models.

	Design	Covariates	Factors	Levels
3-ways full factorial model	*3x2x2*	*age and sex*	group	HC
				non-PS
				PS
			task complexity	TIFs
				TAFs
			movement onset	paced
				unpaced
	*2x2x2*	*age, sex, PANSS total, OLZ and diazepam*	group	non-PS
				PS
			task complexity	TIFs
				TAFs
			movement onset	paced
				unpaced
2-ways full factorial model	*3x4*	*age, sex, PANSS total, OLZ and diazepam*	group	HC
				non-PS
				PS
			conditions	TIF*_paced_*
				TAF*_paced_*
				TIF*_unpaced_*
				TAF*_unpaced_*

TIF*_paced:_* Sound-paced thumb-index finger tapping; TIF*_unpaced_*: unpaced thumb-index finger tapping; TAF*_paced_*: Sound-paced thumb-alternating finger opposition; TAF*_unpaced_*: unpaced thumb-alternating finger opposition.

PS, patients with slowing; non-PS, patients without slowing; HC, healthy controls; OLZ, olanzapin; PANSS, positive and negative syndrome scale.

For each model, we explored the main factor effects and the interaction between them using F-tests.

We applied a cluster-forming threshold of p <.005 and false discovery rate cluster threshold of qFDR <.05. We performed *post-hoc* t-tests when there was a significant interaction or if one significant main factor effect had more than two levels.

To address the issue raised by the small non-PS group, we performed a between-groups comparison in an ROI approach, using the clusters obtained by the HC vs. patients with schizophrenia (merging PS and non-PS) contrasts during any conditions, all comparisons were FDR-corrected. We also correlated the brain activations with both task performance (TIF and TAF_unpaced_) and motor-related clinical scales (SRRS, BFCRS, and UPDRS) using Kendall’s Tau and an FDR correction.

### Generalized psychophysiologic interaction

Additionally, we investigated task-based modulation of functional connectivity across conditions using a generalized psychophysiologic interaction (gPPI) as implemented in the CONN toolbox (v.22a, www.nitrc.org/projects/conn). We followed the toolbox standard preprocessing pipeline with realignment, direct segmentation, and normalization into MNI-space, smoothing with an 8mm FWHM kernel, outlier detection (movement >.9mm, global signal within 97^th^ percentiles), regression of white matter, CSF signal, realignment parameters and scrubbing of outlier volumes, as well as filtering above.008Hz. Then, we modeled a gPPI for the two full factorial designs in a ROI-to-ROI approach with brain areas related to the motor circuit (bilateral pre- and postcentral gyrus, superior parietal lobule (SPL), supplementary motor areas (SMA), dorsal premotor cortex (PMd), anterior cingulate gyrus (ACC), thalamus, caudate, putamen, and pallidum from the Harvard-Oxford atlas (52–55), as well as lobules IV and V of the cerebellum from the AAL atlas (56) as regions of interest. We considered a cluster-level FDR-corrected p<.05 with connection threshold p<.05 to be significant for the gPPI analyses.

## Results

### Clinical characteristics and behavioral performance

As mentioned in the methods section, the vast majority of patients, especially in the PS group, were unable to perform the task accurately after several attempts. The inability to perform the task was present equally in all the conditions. Demographics and differences therein between groups are reported in **Table 1**. The three groups were matched for age and sex and did not differ in these characteristics after exclusions. Both patient groups had fewer years of education than HC. PS had higher PANSS scores than non-PS. As expected and per definition for group allocation, PS had higher ratings in SRRS than the other groups, while non-PS had SRRS ratings in an intermediate position. Similarly, tapping performance in both unpaced conditions was lower in PS than in HC with performance of non-PS in an intermediate position. However, only the differences between PS and HC were significant (**Table 1**).

**Figure 2:**
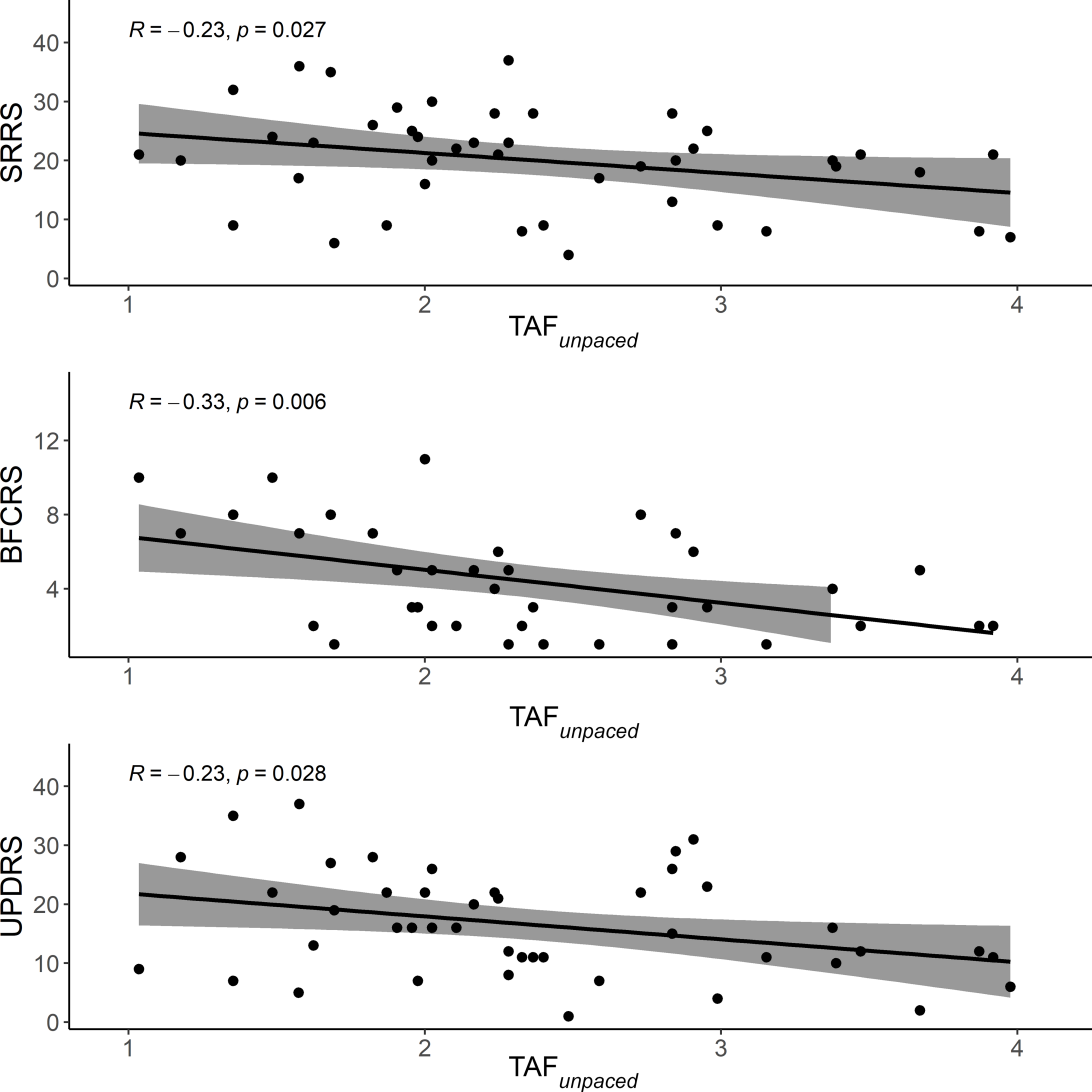
Scatter plot of the correlation between motor-related clinical scales and performance during TAF*_unpaced_*. The solid line is the “line of best fit”, a line that minimizes the vertical distances between the data points and the line itself. The “line of best fit” is a useful way of representing the linear trend. The gray shading around the line represents the 95% confidence interval around the line of best fit. TAF*_unpaced_*, unpaced thumb alternating finger opposition; SRRS, Salpêtrière retardation rating scale; BFCRS, Bush-Francis catatonia rating scale; UPDRS, unified Parkinson’s disease rating scale.

The tapping performance during the TAF*_unpaced_* condition correlated negatively with the three motor-related clinical scales. Better performance was associated with less motor impairment. The strongest correlation was observed with BFCRS (**Figure 2**).

### Task-dependent neural substrates main effects and interactions

**Figure 3:**
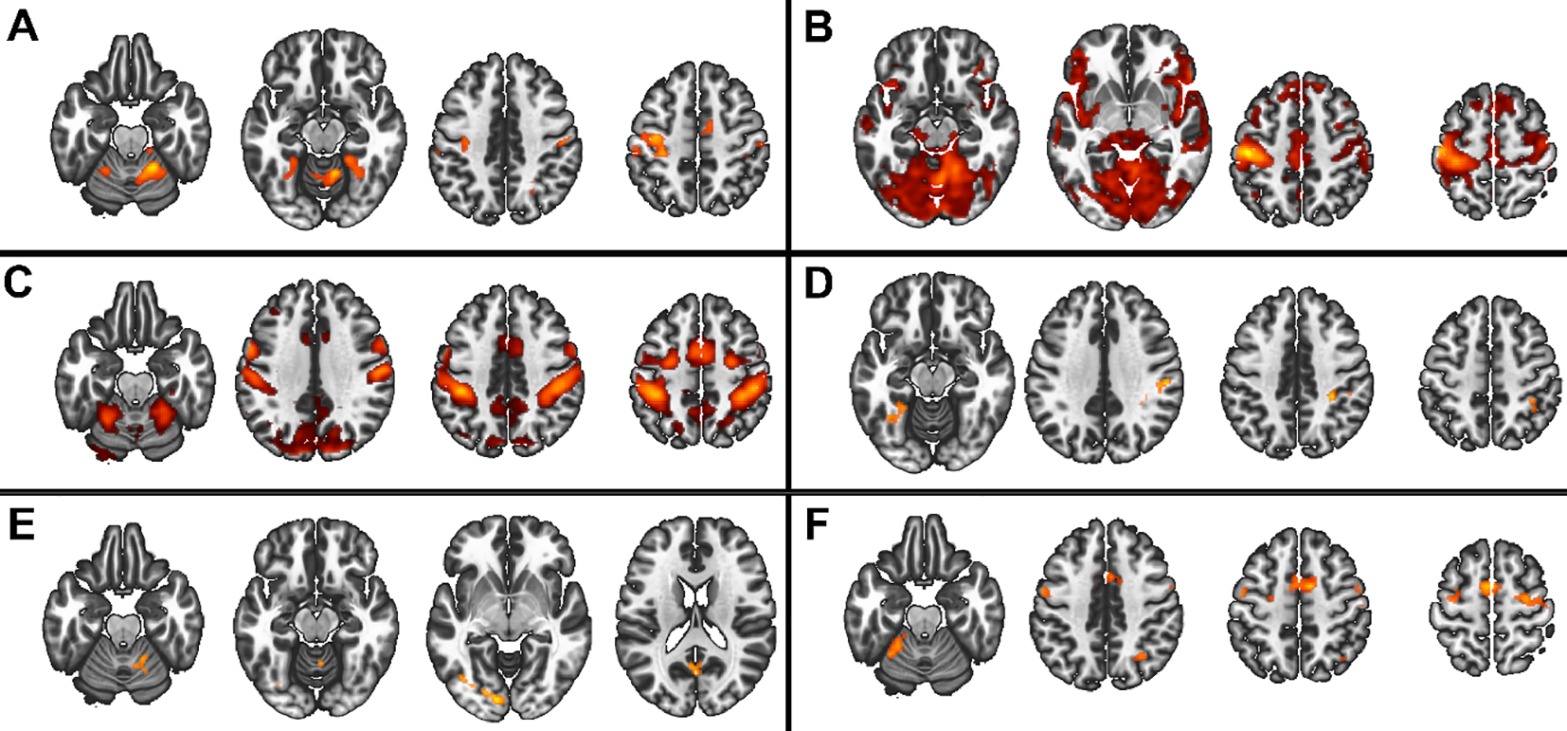
Main group and interaction effects from the 3x2x2 model. Red scale represents the significance of the F-tests. **(A)** Main effect of group, **(B)** Main effect of movement onset, **(C)** Main effect of complexity, **(D)** interaction group by complexity, **(E)** group by onset, **(F)** interaction complexity by movement onset.

In the 3x2x2 design, we found main effects of the three factors (group, task complexity, and movement onset), as well as the following interactions: task complexity by group, movement onset by group, and task complexity by movement onset (**Figure 3**), (all F>5.3 and qFDR<0.005).

**Figure 4:**
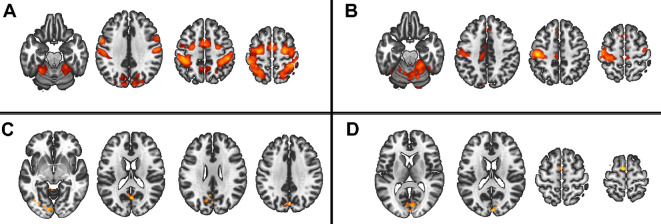
Main group and interaction effect from the 2x2x2 model. Red scale represents the significance of the F-tests. **(A)** Main effect of movement onset, **(B)** Main effect of complexity, **(C)** interaction group by complexity, **(D)** interaction complexity by movement onset.

In the 2x2x2 design, comparing only the two patient groups, we found main effects of task complexity and movement onset factors, and the following interactions: task complexity by group, and, task complexity by movement onset (all F>7.98 and qFDR<0.005), (**Figure 4**).

### Post-hocs

**Figure 5:**
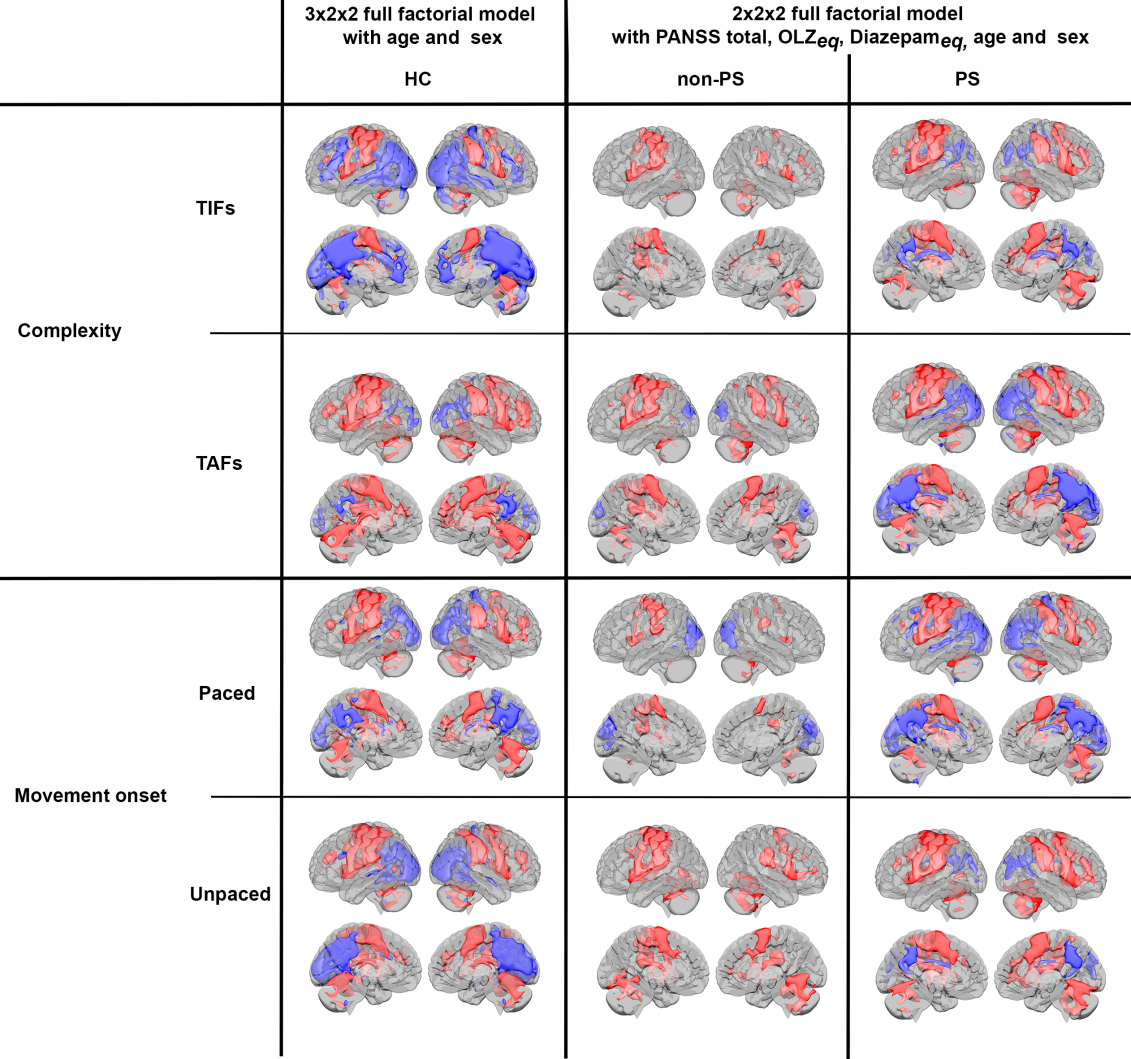
Activations (red) and deactivations (blue) per groups for both complexity and movement onset factor levels. HC, healthy controls; non-PS, non-slowed patients; PS, slowed patients; complexity TIFs: combined TIF*_paced_* and TIF*_unpaced_*; complexity TAFs combined TAF*_paced_* and TAF*_unpaced_*, movement onset paced combined TIF*_paced_* and TAF*_paced_*; movement onset unpaced combined TIF*_unpaced_* and TAF*_unpaced._*. TIF, thumb-index finger tapping; TAF, thumb-alternating finger opposition; eq, equivalent; PANSS, positive and negative syndrome scale; OLZ, olanzapin.

Regardless of the design, all three factors and all levels (**Figure 5**, and **Supplementary Table S1**), showed consistent task-related activations (all t >2.6 and qFDR<0.005) in M1, primary sensory cortex (S1), premotor cortex, SMA, bilateral cerebellum IV-V-VI-VIII, in dorsolateral prefrontal cortex (DLPFC), and SPL, and deactivation in the precuneus (all t < -2.8 and qFDR<0.005).

**Figure 6:**
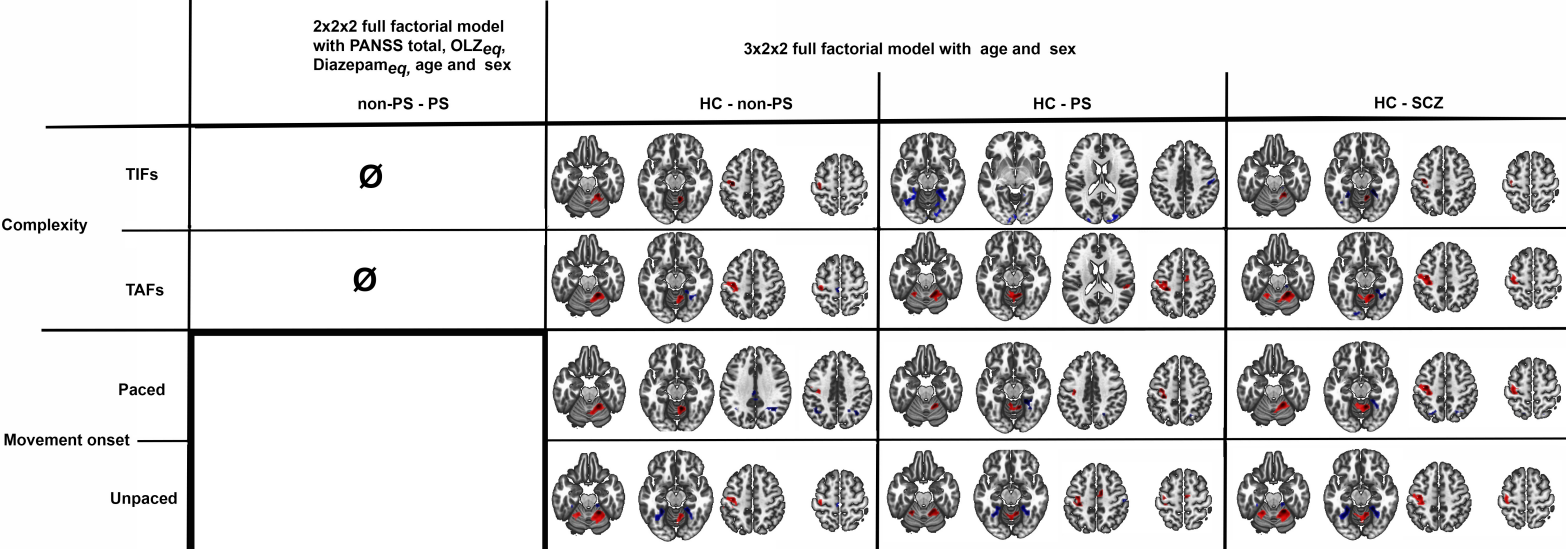
Between groups comparison in whole-brain BOLD response regarding both complexity and movement onset factor levels. Red refers to HC > non-PS, HC> PS, and HC-SCZ. Blue refers to non-PS >HC, PS >HC, and SCZ>HC. HC, healthy controls; non-PS, non-slowed patients; PS, slowed patients; complexity TIFs: combined TIF*_paced_* and TIF*_unpaced_*; complexity TAFs combined TAF*_paced_* and TAF*_unpaced_*, movement onset paced combined TIF*_paced_* and TAF*_paced_*; movement onset unpaced combined TIF*_unpaced_* and TAF*_unpaced._*. TIF, thumb-index finger tapping; TAF, thumb-alternating finger opposition; eq, equivalent; PANSS, positive and negative syndrome scale; OLZ, olanzapin; PS, patients with slowing; non-PS, patients without slowing; HC, healthy controls.

The *post-hoc*s tests of the 3x2x2 design, are detailed in **Figure 6** and **Table 3**. In summary, HC showed higher involvement of M1, S1, and cerebellum IV-V-VI than patients with schizophrenia (HC > SCZ, HC > PS, and HC > non-PS) regardless of task complexity and movement onset levels, while patients with schizophrenia showed higher involvement of SPL, precuneus, and posterior cingulate cortex (PCC) than HC (HC < SCZ, HC < PS, and HC < non-PS), regardless of task complexity and movement onset levels (**Figure 6**, **Table 3**).

**Table 3:** Group difference in task-related brain activation per group.

	Coordinates			cluster size	qFDR	Brain area
HC > SCZ						
Complexity						
*TIFs*	12	-52	-18	297	<0.001	R cer IV-V
	-34	-16	52	240	<0.01	L M1
*TAFs*	-30	-26	48	648	<0.001	L M1, L S1
	16	-50	-20	481	<0.001	B cer IV-V
Movement onset						
*paced*	-34	-16	52	668	<0.001	L M1, L S1
	10	-52	-16	630	<0.001	B cer IV-V, R cer VI
*unpaced*	20	-46	-24	999	<0.001	B cer IV-V-VI
	-36	-14	46	560	<0.001	L M1, L S1
HC < SCZ						
Complexity						
*TIFs*	26	-48	-14	150	<0.05	R fusiform cx
	-24	-50	-12	119	<0.05	L fusiform cx
*TAFs*	6	-64	2	363	<0.001	B V2
	20	-46	-10	287	<0.001	R V2, R fusiform cx
Movement onset						
*paced*	26	-66	28	365	<0.001	R SPL
	-34	-56	38	244	<0.001	L SPL, L IPL
	22	-48	-10	148	<0.05	R V2
	-4	-24	32	138	<0.05	L PCC
*unpaced*	22	-44	-12	383	<0.001	R cer IV-VI
	-24	-48	-12	315	<0.001	R fusiform cx, R cer IV-V
	8	-56	2	267	<0.001	L fusiform cx
HC > non-PS						
Complexity						
*TIFs*	8	-54	-16	318	<0.001	R cer IV-V-VI
	-34	-16	52	226	<0.001	L M1, L S1
*TAFs*	20	-48	-24	546	<0.001	R cer IV-V-VI
	-36	-22	60	492	<0.001	L M1, L S1
Movement onset						
*paced*	-36	-16	48	624	<0.001	L M1, L S1
	8	-54	-16	553	<0.001	R cer IV-V-VI
*unpaced*	20	-48	-24	652	<0.001	R cer IV-V-VI
	-36	-22	60	395	<0.001	L M1, L S1
	-22	-46	-28	190	<0.01	L cer IV-V-VI
	-62	-14	30	95	<0.05	L S1, L IPL
HC < non-PS						
Complexity						
*TAFs*	6	-62	2	370	<0.001	L V2
	20	-44	-10	219	<0.001	R V2, R fusiform cx
	0	-40	38	197	<0.001	PCC
	-4	-62	22	135	<0.05	precuneous
	-2	-24	60	121	<0.05	L M1
	22	-76	-6	113	<0.05	R V2
Movement onset						
*paced*	22	-54	38	276	<0.001	R SPL, R IPL
	-34	-56	28	182	<0.01	L SPL, L IPL
	-4	-26	32	115	<0.05	PCC
*unpaced*	20	-44	-12	267	<0.001	R cer IV-V, R V2, R fusiform cx
	-24	-48	-12	223	<0.01	L fusiform cx
	-8	-38	34	206	<0.01	PCC
	6	-60	2	193	<0.01	precuneous, L V2
	-4	-14	68	95	<0.05	SMA
HC > PS						
Complexity						
*TAFs*	14	-52	-18	627	<0.001	B cer IV-V-VI
	-30	-26	48	587	<0.001	L M1 S1
	54	-32	24	138	<0.01	R M1,R S1, R SPL
	8	-4	48	105	<0.01	SMA
Movement onset						
*paced*	12	-52	-16	332	<0.001	B cer IV-V, R VI
	-28	-28	50	307	<0.001	L M1, L S1
*unpaced*	14	-52	-18	467	<0.001	B cer IV-V, R VI
	-30	-26	48	368	<0.001	L M1, L S1
	10	-2	48	140	<0.05	SMA
	-24	-50	-24	128	<0.05	L cer VI
	54	-34	22	122	<0.05	R IPL
HC < PS						
Complexity						
*TIFs*	20	-40	-16	387	<0.001	R fusiform cx, R cer IV-V
	-32	-56	-16	312	<0.001	L fusiform cx
	28	-88	16	238	<0.01	R fusiform cx, R V2
	-24	-86	12	173	<0.01	L V2
	14	-84	-18	163	<0.01	R fusiform cx
	48	-20	42	143	<0.05	R S1
	-52	-24	52	114	<0.05	L M1
Movement onset						
*paced*	24	-42	-14	206	<0.01	R fusiform cx
*unpaced*	22	-48	-10	250	<0.001	R fusiform cx
	-22	-46	-10	184	<0.01	L fusiform cx
	48	-20	44	153	<0.01	R M1
	-12	-76	6	91	<0.05	L V1

L, left; R, right; B, bilateral; cx, cortex; M1, primary motor cortex; S1, primary sensorimotor cortex; SMA, supplementary motor area; cer, cerebellum; IPL, inferior parietal cortex; SPL, superior parietal cortex; HC, healthy controls; non-PS, non-slowed patients; PS, slowed patients; V1, primary visual cortex; V2, secondary visual cortex; TIF, thumb-index finger tapping; TAF, thumb-alternating finger.

Regarding the 2x2x2 design, we contrasted only group by task complexity as there was no group by movement onset interaction and found no difference between PS and non-PS neither in complexity TIF nor complexity TAF level.

In the 3x4 design, we observed a significant group by conditions interaction (**Supplementary Figure S2**). The *post-hoc* test are displayed in **Supplementary Figure S3**.

**Figure 7:**
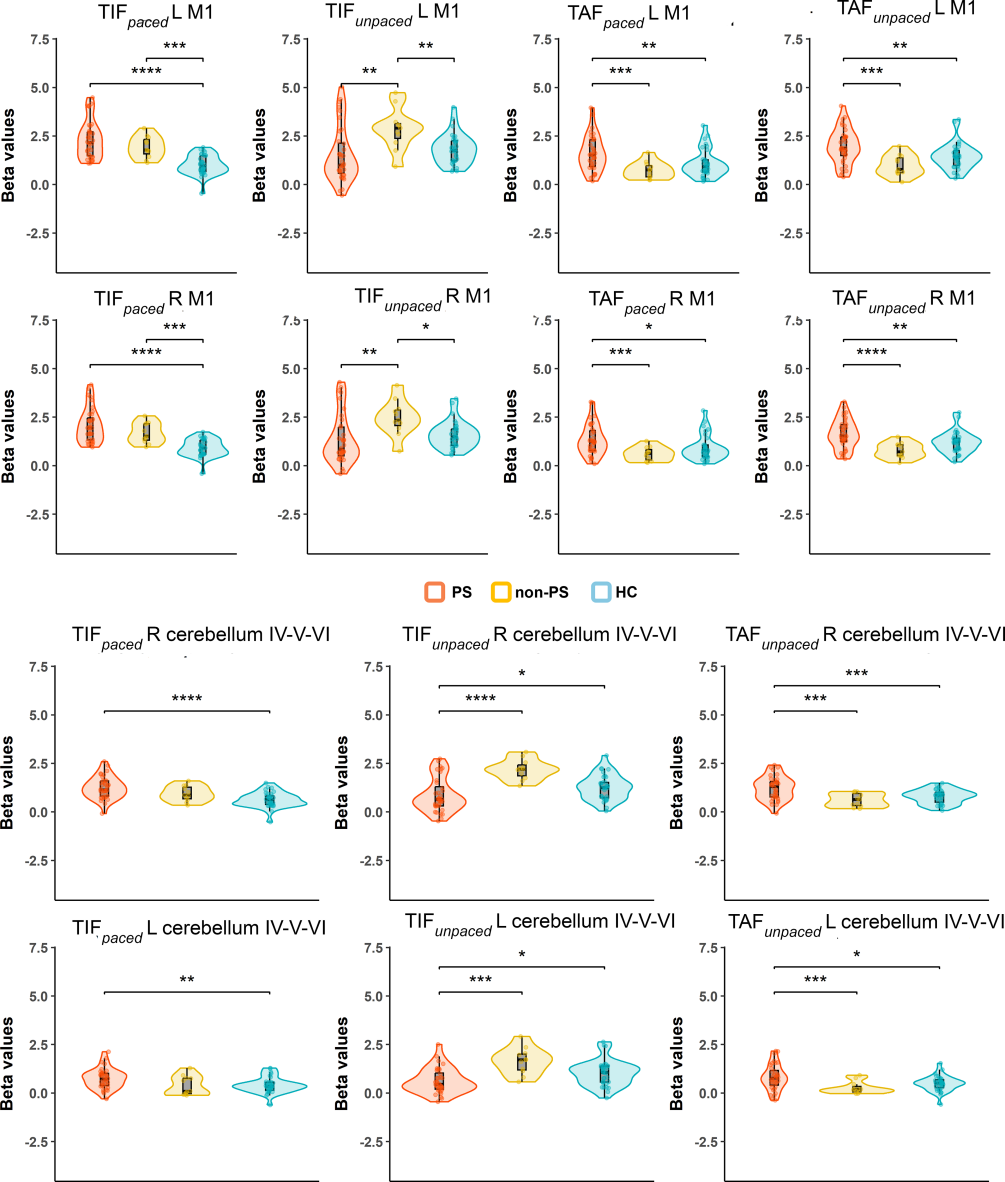
Group by conditions comparison in ROI extracted beta values from significant clusters of HC vs schizophrenia contrasts. The figure displays trimmed violin plots including box-and-whisker plots. The center line represents the median value, the lower bound of the box represents the 25th percentile, the upper bound of the box the 75th percentile, and the whiskers represent 3 times the interquartile range. *p < 0.05, **p < 0.01, ***p < 0.001, ****p< 0.0001. Red is for PS, yellow for non-PS, and blue for HC. R, right; L, left; M1, primary motor cortex; Cerebellum, cluster covering lobules; IV-V-VI HC, healthy controls; non-PS, non-slowed patients; PS, slowed patients; TIF, thumb-index finger tapping; TAF, thumb-alternating finger opposition.

As the group by conditions interaction was significant, we compared group by condition in ROI analyses, we observed higher activation in both patient groups than in HC in bilateral M1 and cerebellum IV-V-VI during the TIF*_paced_* condition. In these regions, during the TIF*_unpaced_* condition, we observed higher activation in non-PS than PS and HC. During the TAF*_paced_* and unpaced condition, the PS group showed higher activation than HC and non-PS in bilateral M1, the same pattern is observed in bilateral cerebellum IV-V but only during the TAF*_unpaced_* conditions (**Figure 7**). Additional brain areas with between-group differences are reported including parietal cortex (SPL, and supramarginal), PCC, or SMA in **Supplementary Figure S4**. Briefly, the group differences in these areas are related to deactivation, in PCC or parietal cortex, PS showed less deactivation than HC, while in SMA, PS showed higher deactivation than HC and non-PS.

### Correlation between tapping performance and brain activation

**Figure 8:**
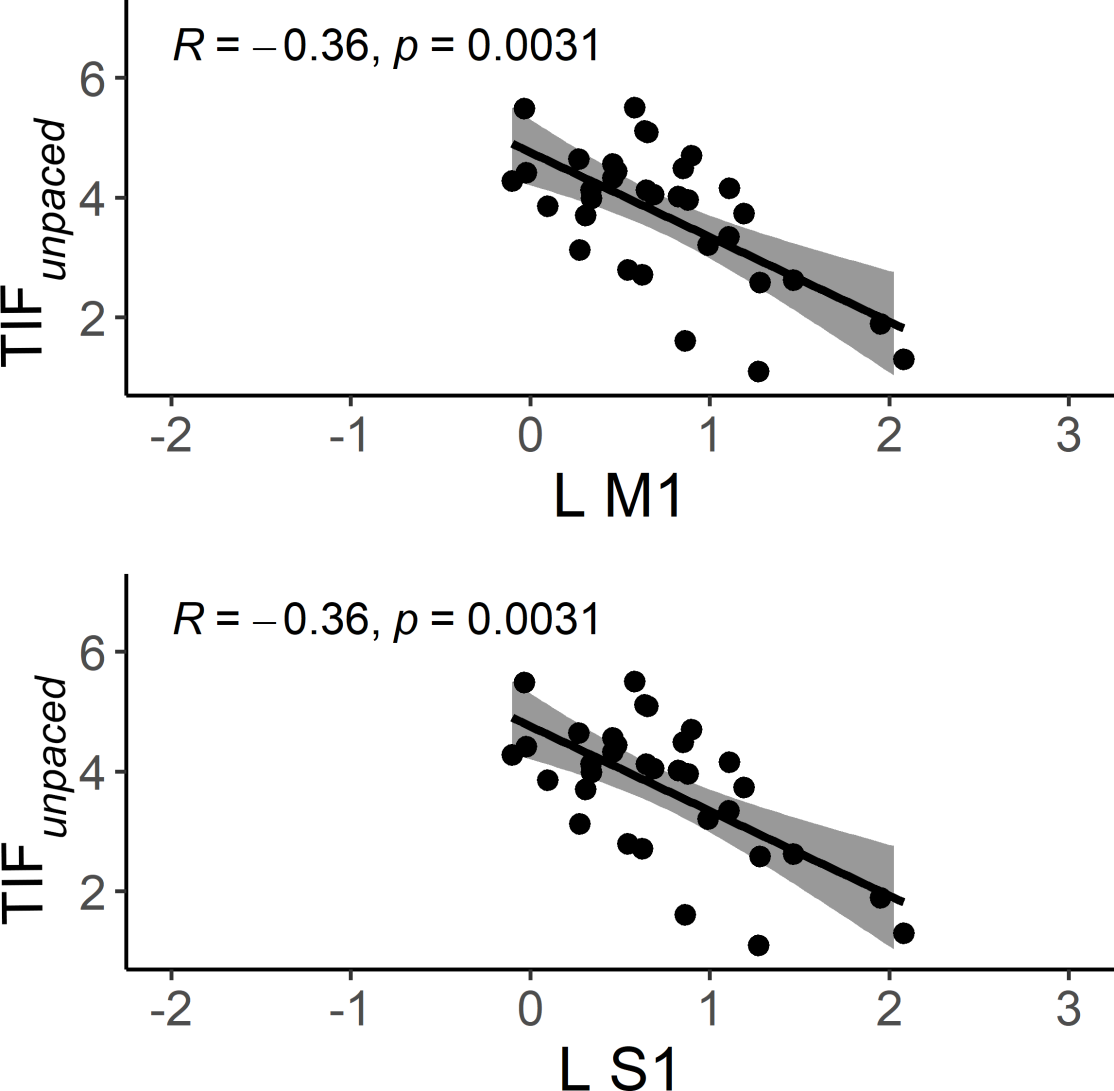
Scatter plots of the correlation between brain activation and performance during TIF and TAF*_unpaced_* conditions in HC. The solid line is the “line of best fit”, a line that minimizes the vertical distances between the data points and the line itself. The “line of best fit” is a useful way of representing the linear trend. The gray shading around the line represents the 95% confidence interval around the line of best fit. L M1, left primary motor cortex; L S1, left primary sensory motor cortex; HC, healthy controls.

In HC, better TIF*_unpaced_* performance is associated with lower activation in left M1 and S1 while we found no association in patients with schizophrenia (merging PS and non-PS) with neither TAF*_unpaced_* nor TIF*_unpaced_* (**Figure 8**).

### Generalized psychophysiologic interaction

The gPPI analyses showed no difference between the three groups.

## Discussion

In this exploratory study, we examined fMRI task responses to finger-tapping in schizophrenia patients with and without psychomotor slowing and healthy controls. Strikingly, about one-half of the schizophrenia patients, mostly patients with psychomotor slowing, were unable to perform this simple motor task correctly. Slowed patients had lower tapping frequency in the unpaced tapping conditions.

Regardless of the task complexity (TIF or TAF) and the movement onset (paced or unpaced) the three groups showed activations in the expected core areas of the motor network including M1, S1, premotor cortex as well as SMA, bilateral cerebellum IV-V-VI-VIII, and DLPFC, and deactivations in precuneus (as in the posterior parts of the DMN (57, 58)). We found group differences in whole-brain task-based neural substrates: i) higher involvement of M1, S1, and cerebellum IV-V-VI in HC than patients in all the task complexity and movement onset factor levels, ii) patients with schizophrenia showed higher involvement of SPL, precuneus, and posterior cingulate cortex (PCC) than HC in all the task complexity and movement onset factor levels.

At the ROI level, the role of the cerebellum appears to be more complex. While its activation remains fairly constant across all conditions in the PS group, the other two groups seem to adjust the cerebellum’s involvement based on the specific task. This study extends prior work by focusing on patients with severe psychomotor slowing specifically (PS) (49).

Half of PS being unable to perform this finger-tapping task correctly regardless of the condition, and the other one being less performant than healthy controls and non-PS, reflects the huge impairment of these patients in their daily life as well as the higher global severity of the disease observed in this group. These findings are in line with the poor outcomes associated with psychomotor slowing (8) (4–7). Despite the lack of difference in the medication dosage between the patients with and without psychomotor slowing, one could argue that these differences in performance could be a side effect of medication. Motor abnormalities including psychomotor slowing have long been exclusively linked to medication side effects; however, converging evidence demonstrates that motor abnormalities frequently occur before medication is commenced and even long before the onset of psychosis (14). These observations need to be confirmed in future studies with an extended number of non-PS patients.

In line with our hypothesis, the tapping speed in the unpaced tapping conditions was lowest in the slowed patients. This indicates that psychomotor slowing as defined using the SRRS does reflect deficits in fine motor finger movements. While the non-slow patients took an intermediate position in terms of tapping frequency, differences to the other groups were not significant, possibly due to the small sample size of the non-PS group. Moreover, the tapping performance is associated with the severity of motor abnormalities. This is consistent with prior results in coin rotation tasks, another measure of fine motor behaviour (9). Deficits in finger tapping have even been observed in first-degree relatives of patients with schizophrenia, suggesting that poor finger-tapping performance may serve as a marker of vulnerability for schizophrenia (59).

At the whole-brain level, HC showed higher activity than schizophrenia patients, regardless of complexity and movement onset factor levels, in the bilateral cerebellum IV-V, M1 and S1, while schizophrenia patients showed higher activity in the SPL. We performed a deeper exploration of the involvement of these brain regions in an ROI analysis. We observed that despite the condition-based regulation of bilateral M1s’ involvement between the three groups, the activation of bilateral M1s seems to be higher in PS than the two other groups except for the TIF*_unpaced_* condition where non-PS have higher activation than the two other groups. The involvement of the cerebellum seems more nuanced: the recruitment of the cerebellum during this finger tapping task in PS, is not modulated by the conditions in opposite to HC and non-PS. This may result from aberrant integration of the cerebellum into motor circuits (60). Previous research linked resting-state dysconnectivity of the cerebellum to poor performance during finger tapping or pegboard tasks in psychosis in general (32, 61). Our gPPI analysis failed to demonstrate group differences in task-related functional connectivity changes, while previous research suggested an altered effective cerebello-cerebral connectivity during finger tapping in patients with schizophrenia (62). Classically, the cerebellum has been strongly associated with motor function, specifically during online error correction when movement plans are compared to proprioceptive feedback. Lesions in the cerebellum can cause ataxia and coordination impairments (63). Moreover, the cerebellar timing hypothesis suggests that the cerebellum has internal timing functions (64–66). Therefore, unmodulated cerebellum activation during finger tapping in schizophrenia may impair tapping performance via altered error correction, coordination, or impaired internal timing.

While we found SPL to be deactivated in most of the conditions in HC and non-PS, about half of slowed patients activated the SPL instead of deactivating it. This may be related to the role of the SPL in multimodal integration and spatial processing (67, 68). In fact, altered SPL neuroimaging measures are related to deficits in manual movements such as gesturing in schizophrenia (69–73). Therefore, the activation of SPL in half of PS possibly represents the recruitment of additional neural resources to compensate for the alteration of cerebellar function.

We found no differences between groups regarding task-associated functional connectivity. These psychophysical interactions can be hard to detect, and our sample might not have had sufficient power to do so. Schizophrenia is a very heterogeneous disorder and although we removed some of that variability by grouping patients according to the presence of psychomotor slowing, there might still be considerable heterogeneity in functional connectivity in the patient groups. An alternative explanation for these null findings could be that the integration of multiple signals across the motor circuitry is impaired in schizophrenia, aligning with prior findings of aberrant cortical excitability and cerebellar connectivity in patients with psychomotor slowing in psychosis (33). Likewise, cerebellar-prefrontal connectivity is linked to psychomotor performance, particularly slowing (32). These findings speak towards poor cerebellar-cerebral connectivity in psychosis, which seems to be specific to patients with PS.

Several limitations need to be considered for the interpretation of this study. First, we had to exclude a substantial proportion of patients due to insufficient performance and quality issues (PS: 28%, non-PS: 31%), decreasing sample sizes. Consequently, the non-PS group (N=11) was very small, lacking sufficient statistical power to detect differences compared to the other groups. Next, the task consisted of a limited number of trials for each condition. However, we found acceptable reliability for this task implementation in HC (49). Finally, we recorded daily medications but lacked information on total antipsychotic and benzodiazepine exposure. However, accounting for potential effect of current medication dosage (OLZ*_eq_*., diaz*_eq_*) we included medication as a covariate in the patient groups comparison.

## Conclusion

Slowed patients with schizophrenia show poorer finger-tapping performance that is paralleled by the inability to modulate cerebellar activity and insufficient deactivation of the superior parietal lobule compared with HC. Half of the patients with schizophrenia recruited additional neural resources in response to the task, but this did not compensate for the poor performance.

## Data Availability

The raw data supporting the conclusions of this article will be made available by the authors, without undue reservation.
