# Supplementary figures and images for: Reduced finger tapping speed in patients with schizophrenia and psychomotor slowing: an exploratory fMRI study
Source: Front Psychiatry. 2025 Apr 28;16:1539112. doi: 10.3389/fpsyt.2025.1539112 (PMC12066633; doi:10.3389/fpsyt.2025.1539112)

Figure S1: Participant flow diagram


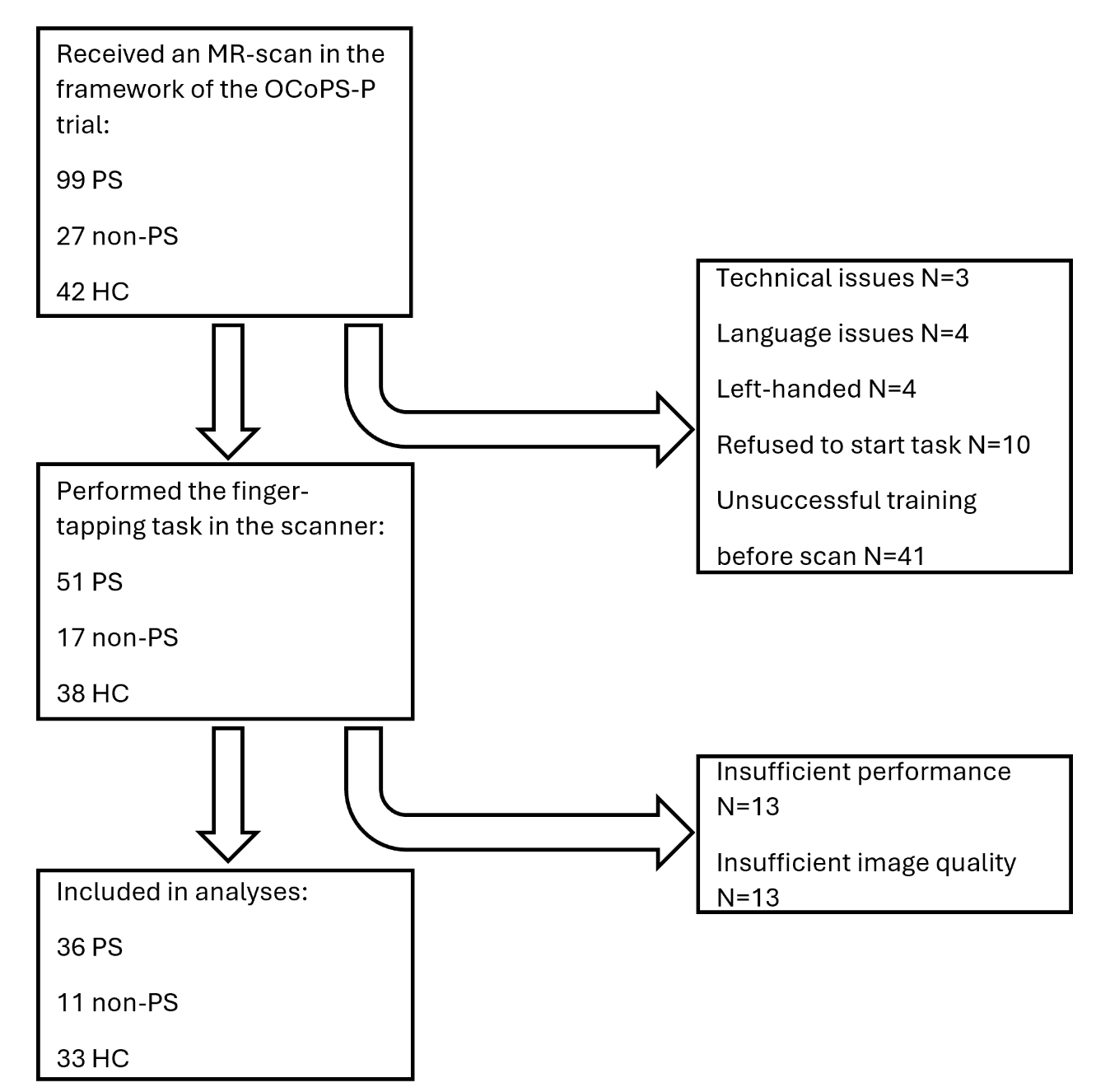

Supplement: Supplementary file 1 [file SupplementaryFile1.docx]
