# Supplementary material for: Reduced finger tapping speed in patients with schizophrenia and psychomotor slowing: an exploratory fMRI study
Source: Front Psychiatry. 2025 Apr 28;16:1539112. doi: 10.3389/fpsyt.2025.1539112 (PMC12066633; doi:10.3389/fpsyt.2025.1539112)
Supplement: Supplementary file 2 [file SupplementaryFile2.docx]

**Supplementary Figure S2: Activations (red) and deactivations (blue) in all the conditions per group**.

**
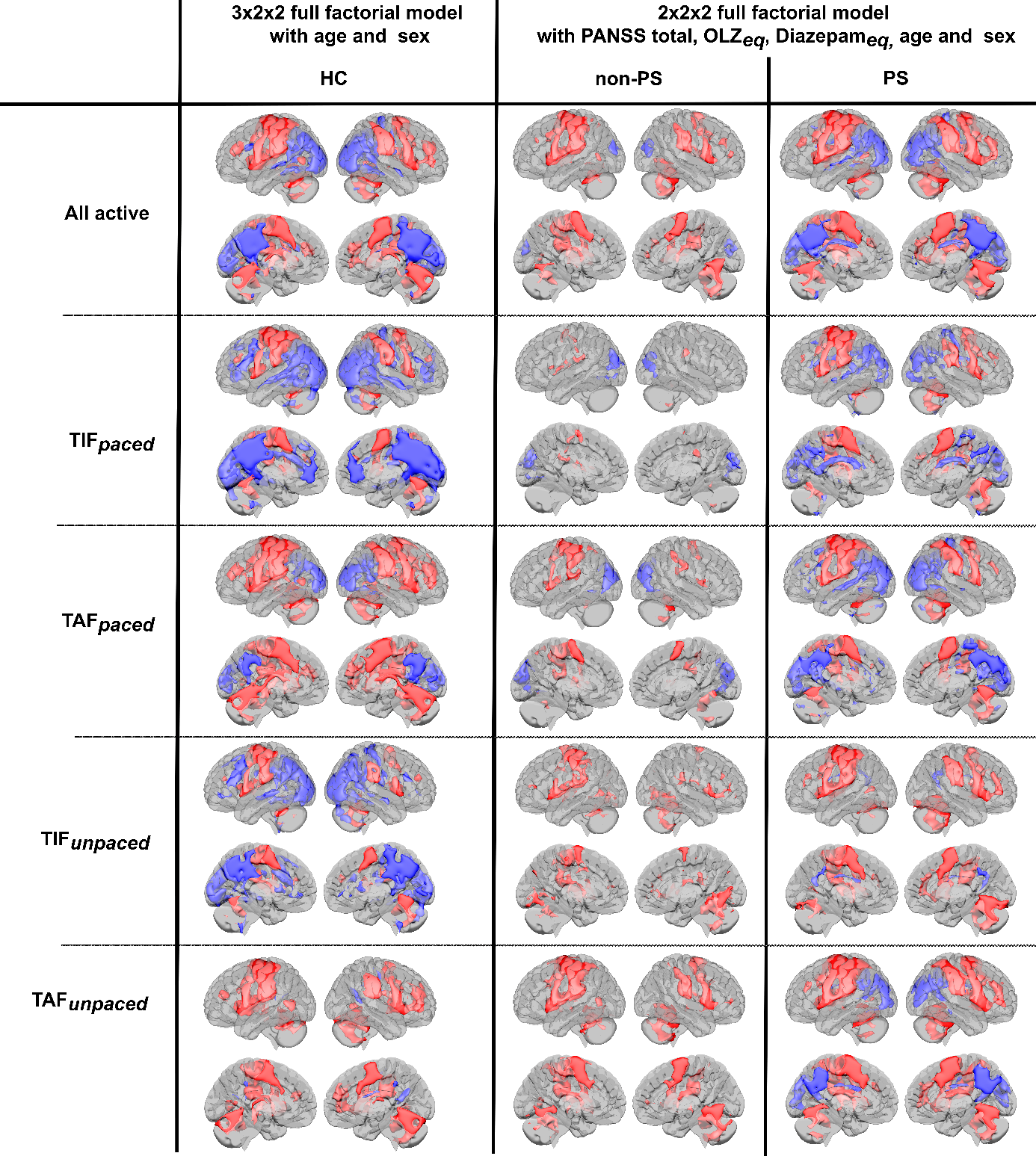
**

HC: healthy controls, non-PS: non-slowed patients, PS: slowed patients, complexity TIFs: combined TIF*_paced_* and TIF*_unpaced_*; complexity TAFs combined TAF*_paced_* and TAF*_unpaced_*, movement onset paced combined TIF*_paced_* and TAF*_paced_*; movement onset unpaced combined TIF*_unpaced_* and TAF*_unpaced._*
