# Supplementary material for: Reduced finger tapping speed in patients with schizophrenia and psychomotor slowing: an exploratory fMRI study
Source: Front Psychiatry. 2025 Apr 28;16:1539112. doi: 10.3389/fpsyt.2025.1539112 (PMC12066633; doi:10.3389/fpsyt.2025.1539112)
Supplement: Supplementary file 3 [file SupplementaryFile3.docx]

Figure S3: **Between groups comparison in whole-brain BOLD response during all the conditions.**
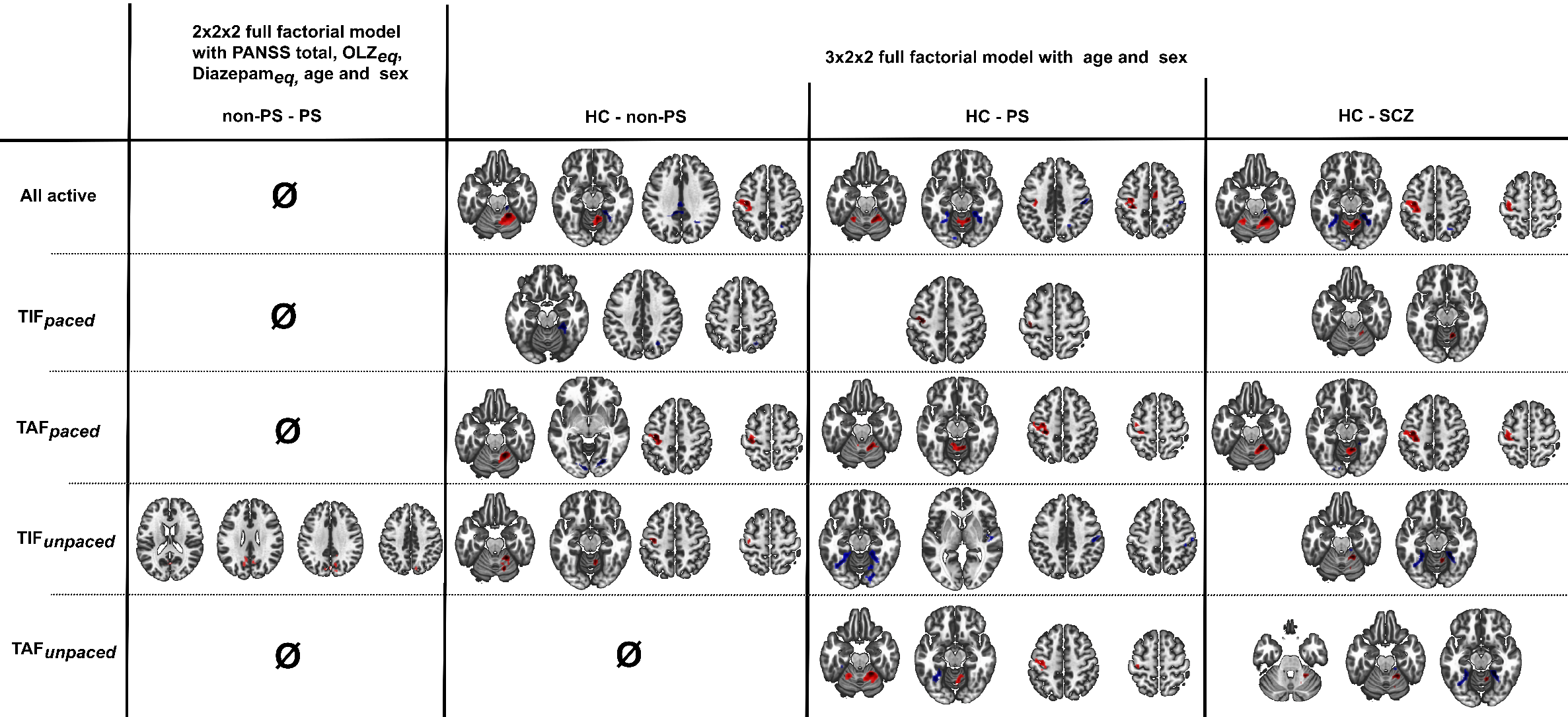


HC: healthy controls; non-PS: non-slowed patients; PS: slowed patients; complexity TIFs: combined TIF*_paced_* and TIF*_unpaced_*; complexity TAFs combined TAF*_paced_*
