# Supplementary material for: Reduced finger tapping speed in patients with schizophrenia and psychomotor slowing: an exploratory fMRI study
Source: Front Psychiatry. 2025 Apr 28;16:1539112. doi: 10.3389/fpsyt.2025.1539112 (PMC12066633; doi:10.3389/fpsyt.2025.1539112)
Supplement: Supplementary file 4 [file SupplementaryFile4.docx]

Figure S4:Additional ROIs extracted beta values group comparison.
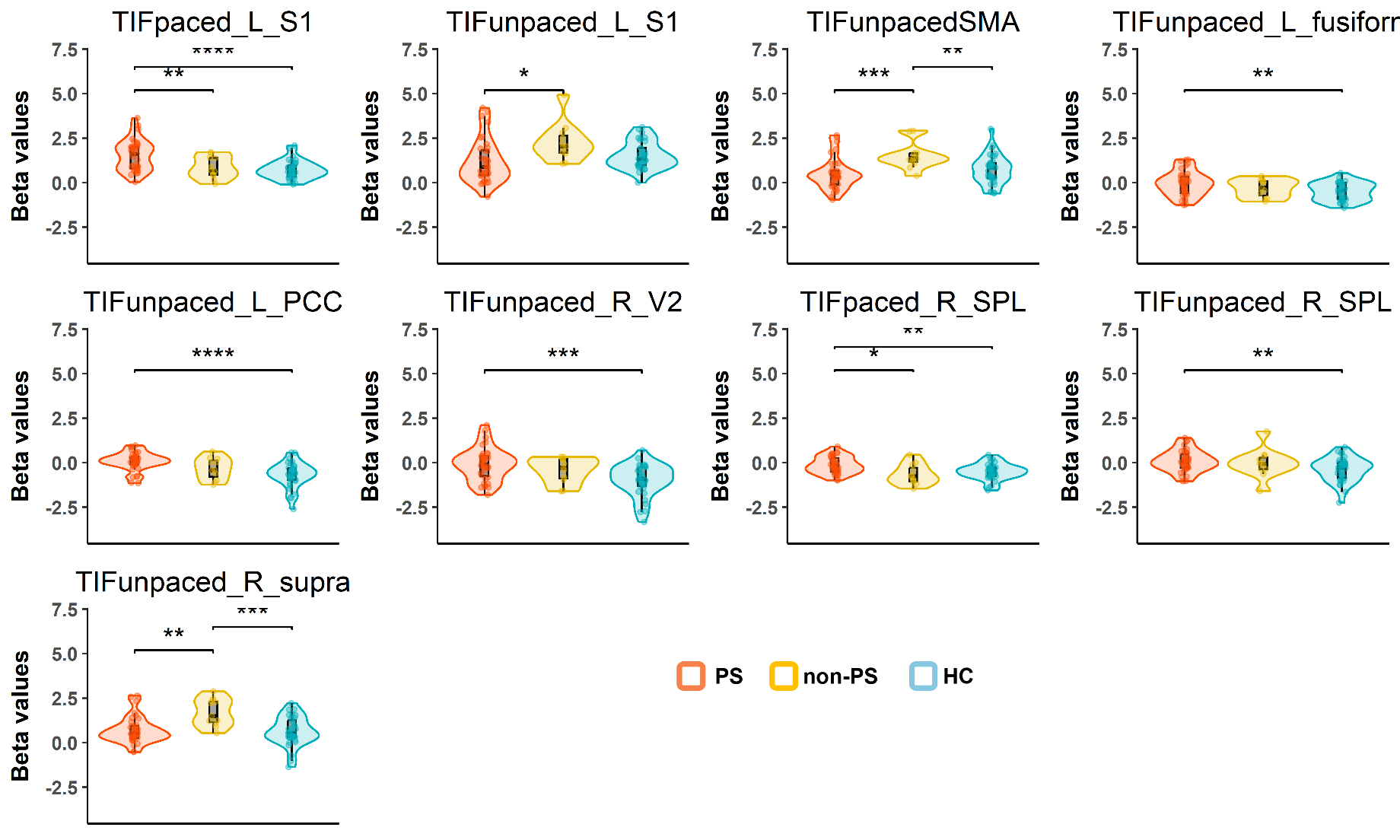


The figure displays trimmed violin plots including box-and-whisker plots. The center line represents the median value, the lower bound of the box represents the 25th percentile, the upper bound of the box the 75th percentile, and the whiskers represent 3 times the interquartile range. **p* < 0.05, ***p* < 0.01, ****p* < 0.001. Red is for PS, yellow for non-PS, and blue for HC.
R: right, L: left, M1: primary motor cortex, HC: healthy controls; non-PS: non-slowed patients; PS: slowed patients.
