# Supplementary material for: Reduced finger tapping speed in patients with schizophrenia and psychomotor slowing: an exploratory fMRI study
Source: Front Psychiatry. 2025 Apr 28;16:1539112. doi: 10.3389/fpsyt.2025.1539112 (PMC12066633; doi:10.3389/fpsyt.2025.1539112)
Supplement: Supplementary file 5 [file Table1.docx]

**Table S1: Task-related brain (de)activation per group**

| **HC**  **Complexity** | | | | | | | | | | | | | | | | | | | | | | | | | |  |  |
| --- | --- | --- | --- | --- | --- | --- | --- | --- | --- | --- | --- | --- | --- | --- | --- | --- | --- | --- | --- | --- | --- | --- | --- | --- | --- | --- | --- |
|  |  |  |  |  |  |  |  |  |  |  |  |  |  |  |  |  |  |  |  |  |  |  |  |  |  |  |  |
| ***Activation TIFs*** | | -34 | | | | | | | | -22 | | | | | 52 | 8703 | | | | | | <0.001 | | L pre/postCG, SMA,L SPL, B CC | |  |  |
|  | | 20 | | | | | | | | -48 | | | | | -22 | 3272 | | | | | | <0.001 | | R cer IV-V-VI-VIII | |  |  |
|  | | 54 | | | | | | | | -20 | | | | | 32 | 2528 | | | | | | <0.001 | | R IPL, R CC, R SPL, | |  |  |
|  | | -44 | | | | | | | | 0 | | | | | 8 | 2244 | | | | | | <0.001 | | L preCG, L IC, L put, L FC | |  |  |
|  | | 44 | | | | | | | | 2 | | | | | 8 | 1803 | | | | | | <0.001 | | R preCG, R IC, R put, R FC | |  |  |
|  | | -26 | | | | | | | | -52 | | | | | -24 | 959 | | | | | | <0.001 | | L cer IV-V-VI | |  |  |
| ***Deactivation TIFs*** | | -26 | | | | | | | | -88 | | | | | 12 | 32628 | | | | | | <0.001 | | B precuneous, B cuneus, B IPL, B V2, B CC, B LG | |  |  |
|  | | -48 | | | | | | | | 22 | | | | | 26 | 4179 | | | | | | <0.001 | | L FC, L CC | |  |  |
|  | | 60 | | | | | | | | -4 | | | | | -14 | 783 | | | | | | <0.001 | | R temp cx | |  |  |
|  | | 48 | | | | | | | | 22 | | | | | 24 | 516 | | | | | | <0.001 | | R FC | |  |  |
|  | | -6 | | | | | | | | -44 | | | | | -46 | 428 | | | | | | <0.001 | | L cer IX | |  |  |
| ***Activation TAFs*** | | 18 | | | | | | | | -48 | | | | | -20 | 33783 | | | | | | <0.001 | | B pre/postCG, SMA, L SPL, B CC, B FC, B cer IV-V-VI-VIII, B IC, L SPL, B thal, L IPL, B put, B FC,  R temp, R caudate | |  |  |
|  | | 58 | | | | | | | | -32 | | | | | 22 | 3255 | | | | | | <0.001 | | R postCG, R SPL, R IPL | |  |  |
|  | | -46 | | | | | | | | -66 | | | | | 10 | 441 | | | | | | <0.001 | | L temp cx | |  |  |
|  | | 56 | | | | | | | | -58 | | | | | 6 | 254 | | | | | | <0.001 | | R temp cx | |  |  |
| ***Deactivation TAFs*** | | 16 | | | | | | | | -48 | | | | | 10 | 3737 | | | | | | <0.001 | | Precuneous, B CC, R fusiform cx, R V2 | |  |  |
|  | | -38 | | | | | | | | -82 | | | | | 22 | | 1027 | | | | | <0.001 | | L V2 | | |  |
| **HC** | | | | | | | | | | | | | | | | | | | | | | | | | |  |  |
| **Movement onset** | | | | | | | | | | | | | | | | | | | | | | | | | |  |  |
| ***Activation paced*** | | -34 | | | | | -22 | | | | | 52 | | | | | 12970 | | | <0.001 | | | | L pre/postCG, SMA,L SPL, L CC, L IPL |  |  |  |
|  | | 18 | | | | | -48 | | | | | -20 | | | | | 6121 | | | <0.001 | | | | B cer IV-V-VI | |  |  |
|  | | 44 | | | | | 2 | | | | | 8 | | | | | 4108 | | | <0.001 | | | | R FC, R preCG, R IC, R FC, R put, R thal R caudate | |  |  |
|  | | 58 | | | | | | | | -32 | | | | | 22 | 1817 | | | | <0.001 | | | | R IPL, R postCG, | |  |  |
|  | | -28 | | | | | | | | 38 | | | | | 22 | 536 | | | | <0.001 | | | | L FC | |  |  |
|  | | -52 | | | | | | | | -60 | | | | | 6 | 420 | | | | <0.001 | | | | L temp cx | |  |  |
| ***Deactivation paced*** | | 14 | | | | | | | | -56 | | | | | 26 | 11435 | | | | <0.001 | | | | Precuneous, B CC, R fusiform cx, B V2 | |  |  |
|  | | 26 | | | | | -30 | | | | | | | | -16 | | 145 | | | <0.05 | | | | R parahippocampus | |  |  |
| ***Activation unpaced*** | | 20 | | | | | -48 | | | | | | | | -22 | | 31287 | | | <0.001 | | | | B pre/postCG, SMA,B SPL, B CC, B FC, B cer IV-V-VI-VIII, B IC, B thal, B SMG, B put,B FC | |  |  |
|  | | | 34 | | | | | 40 | | | | | 24 | | | | 1086 | | <0.001 | | | | | R FC | |  |  |
|  | | | -30 | | | | | 38 | | | | | 24 | | | | 713 | | <0.001 | | | | | L FC | |  |  |
|  | | | -14 | | | | | -64 | | | | | -48 | | | | 311 | | <0.001 | | | | | L cer VIII-IX | |  |  |
| ***Deactivation unpaced*** | | | 34 | | | | | -84 | | | | | 18 | | | | 22375 | | <0.001 | | | | | Precuneus, BCC, B IPL, B V2 | |  |  |
|  | | | -50 | | | | | 20 | | | | | 28 | | | | 408 | | <0.001 | | | | | L FC | |  |  |
|  | | | -58 | | | | | -36 | | | | | 2 | | | | 332 | | <0.001 | | | | | L temp cx | |  |  |
|  |  |  | -2 | | | | | -50 | | | | | -48 | | | | 176 | | <0.05 | | | | | R cer IX | |  |  |
|  |  |  | 30 | | | | | -74 | | | | | -40 | | | | 102 | | <0.05 | | | | | R cer CRUS I-II | |  |  |
| **non-PS** | | | | | | | | | | | | | | | | | | | | | | | | | |  |  |
| **Complexity** | | | | | | | | | | | | | | | | | | | | | | | | | |  |  |
| ***Activation TIFs*** | | | | -40 | | | | | -24 | | | | | 50 | | | 8421 | | | | <0.001 | | L pre/postCG cx, SMA, L IPL, L put, L IC, L CC, L temp cx | | |  |  |
|  | | | | 18 | | | | | -46 | | | | | -20 | | | 2991 | | | | <0.001 | | R cer IV-V-VI-VIII | | |  |  |
|  | | | | 40 | | | | | 6 | | | | | 4 | | | 1224 | | | | <0.001 | | R Broca area, R put, R IC, R FC | | |  |  |
|  | | | | 52 | | | | | -32 | | | | | 22 | | | 1119 | | | | <0.001 | | R IPL, R SPL | | |  |  |
|  | | | | -34 | | | | | -50 | | | | | -30 | | | 661 | | | | <0.001 | | L cer IV-V-VI | | |  |  |
|  | | | | 44 | | | | | 34 | | | | | 30 | | | 639 | | | | <0.001 | | R PF | | |  |  |
|  | | | | -46 | | | | | -66 | | | | | -2 | | | 192 | | | | <0.05 | | L fusiform cx | | |  |  |
| ***Activation TAFs*** | | | | -32 | | | | | -14 | | | | | 66 | | | 12251 | | | | <0.001 | | B pre/postCG, B SPL, SMA, B CC, L temp cx, L FC, B thal, B put, B IPL, L caudate | | |  |  |
|  | | | | 22 | | | | | -42 | | | | | -22 | | | 3697 | | | | <0.001 | | R cer IV-V-VI- VIII | | |  |  |
|  | | | | 54 | | | | | -16 | | | | | 32 | | | 2255 | | | | <0.001 | | R preCG, R FC, R IC | | |  |  |
|  | | | | 50 | | | | | 10 | | | | | 6 | | | 1785 | | | | <0.001 | | R Broca area | | |  |  |
|  | | | | -32 | | | | | -48 | | | | | -30 | | | 846 | | | | <0.001 | | R cer IV-V-VI | | |  |  |
|  | | | | -46 | | | | | -60 | | | | | 10 | | | 140 | | | | <0.05 | | L OC, L temp cx | | |  |  |
|  | | | | 24 | | | | | -56 | | | | | 52 | | | 123 | | | | <0.05 | | R OC, R SPL | | |  |  |
|  | | | | 38 | | | | | 52 | | | | | 10 | | | 80 | | | | <0.05 | | L thal | | |  |  |
| ***Deactivation TAFs*** | | | | 30 | | | | | -82 | | | | | 32 | | | 875 | | | | <0.001 | | R OC, R cuneus | | |  |  |
|  | | | | -34 | | | | | -90 | | | | | 12 | | | 816 | | | | <0.001 | | L OC, L cuneus | | |  |  |
| **non-PS** | | | | | | | | | | | | | | | | | | | | | | | | | |  |  |
| **Movement onset** | | | | | | | | | | | | | | | | | | | | | | | | | |  |  |
| **Activation paced** | | | | -38 | | | | | 0 | | | | | 8 | | | 1188 | | | | <0.001 | | L pre/postCG, L SPL, SMA,L IPL, L put, L IC, L CC, L temp cx, L FC, L thal | | |  |  |
|  | | | | 20 | | | | | -48 | | | | | -20 | | | 711 | | | | <0.001 | | R IC, R preCG, R put, R Broca area, R FC | | |  |  |
|  | | | | 52 | | | | | -18 | | | | | 32 | | | 524 | | | | <0.001 | | R cer IV-V-VI | | |  |  |
|  | | | | 30 | | | | | -48 | | | | | -52 | | | 349 | | | | <0.001 | | R cer VIII-IX | | |  |  |
|  | | | | 44 | | | | | 8 | | | | | 4 | | | 331 | | | | <0.001 | | R IC, R Broca area | | |  |  |
|  | | | | 50 | | | | | -38 | | | | | 56 | | | 232 | | | | <0.001 | | R IPL, R SPL, R PostCG | | |  |  |
|  | | | | -34 | | | | | -48 | | | | | -30 | | | 133 | | | | <0.05 | | L cer VI | | |  |  |
| **Deactivation paced** | | | | -34 | | | | | -88 | | | | | 14 | | | 6278 | | | | <0.001 | | B IPL, B V2, B cuneous, Precuneous, | | |  |  |
|  | | | | -28 | | | | | -60 | | | | | -8 | | | 117 | | | | <0.05 | | L LG, L fusiform cx | | |  |  |
| **Activation unpaced** | | | | -32 | | | | | -14 | | | | | 66 | | | 12546 | | | | <0.001 | | L pre/postCG, L SPL, L IPL, SMA, L put, L IC, L CC, L temp cx, L FC, L thal, L caudate | | |  |  |
|  | | | | 22 | | | | | -42 | | | | | -22 | | | 5072 | | | | <0.001 | | R cer IV-V-VI-VIII, R LG, R fusiform cx | | |  |  |
|  | | | | | 54 | | | | | | 8 | | | 6 | | | 2185 | | | | <0.001 | | R preCG, R FC, R IC, R put | | |  |  |
|  | | | | | -34 | | | | | | -48 | | | -30 | | | 1347 | | | | <0.001 | | L cer IV-V-VI, L fusiform cx | | |  |  |
|  | | | | | 56 | | | | | | -32 | | | 22 | | | 1291 | | | | <0.001 | | R IPL, R postCG | | |  |  |
|  | | | | | -46 | | | | | | -62 | | | 10 | | | 381 | | | | <0.001 | | L fusiform cx, L temp cx | | |  |  |
|  | | | | | 42 | | | | | | 34 | | | 30 | | | 111 | | | | <0.05 | | R PFC | | |  |  |
| **PS** |  | | | | |  | | | | | | | |  | | |  |  | | | | |  | | |  |  |
| **Complexity** |  | | | | |  | | | | | | | |  | | |  |  | | | | |  | | |  |  |
| **Activation TIFs** | | | | | -34 | | | | | | -22 | | | 52 | | | 18233 | | | | <0.001 | | B pre/postCG, SMA, L CC, L temp cx, L FC | | |  |  |
|  | | | | | 16 | | | | | | -46 | | | -20 | | | 6492 | | | | <0.001 | | R cer IV-V-VI-VIII, B LG, B fusiform cx | | |  |  |
|  | | | | | 58 | | | | | | -20 | | | 22 | | | 2505 | | | | <0.001 | | R postCG, R IPL, R SPL | | |  |  |
|  | | | | | -40 | | | | | | 34 | | | 32 | | | 296 | | | | <0.001 | | L FC | | |  |  |
| **Deactivation TIFs** | | | | | -8 | | | | | | -64 | | | 20 | | | 2017 | | | | <0.001 | | precuneus, CC | | |  |  |
|  | | | | | 16 | | | | | | -84 | | | 36 | | | 727 | | | | <0.001 | | R V2 | | |  |  |
|  | | | | | -20 | | | | | | -84 | | | 36 | | | 284 | | | | <0.001 | | L V2 | | |  |  |
|  | | | | | -46 | | | | | | -64 | | | 26 | | | 398 | | | | <0.001 | | L IPL | | |  |  |
|  | | | | | 46 | | | | | | -54 | | | 24 | | | 374 | | | | <0.001 | | R IPL | | |  |  |
| **Activation TAFs** | | | | | -36 | | | | | | -24 | | | 56 | | | 25103 | | | | <0.001 | | B pre/postCG, B SPL, SMA, B CC, L temp cx, L FC, B thal, B put, B IPL, L caudate | | |  |  |
|  | | | | | 18 | | | | | | -46 | | | -22 | | | 6944 | | | | <0.001 | | B cer IV-V-VI, R LG, R fusiform cx | | |  |  |
|  | | | | | -20 | | | | | | -54 | | | -48 | | | 359 | | | | <0.001 | | L cer VIII-IX | | |  |  |
|  | | | | | -34 | | | | | | 34 | | | 28 | | | 336 | | | | <0.001 | | L FC | | |  |  |
| **Deactivation TAFs** | | | | | 38 | | | | | | -84 | | | 12 | | | 15843 | | | | <0.001 | | precuneous, B cuneus, B V2, B CC, B IPL, B fusiform cx | | |  |  |
|  | | | | | -56 | | | | | | 2 | | | -16 | | | 544 | | | | <0.001 | | L temp cx | | |  |  |
|  | | | | | 20 | | | | | | -76 | | | -36 | | | 241 | | | | <0.001 | | R cer CRUS I-II | | |  |  |
|  | | | | | 40 | | | | | | -14 | | | 42 | | | 239 | | | | <0.001 | | R pre/postCG | | |  |  |
| **PS**  **Movement onset** | | | | | | | | | | | | | | | | | | | | | | | | | |  |  |
| **Activation paced** | | | | | 16 | | | | | | -46 | | | -20 | | | 4323 | | | | <0.001 | | R cer IV-V-VI-VIII, R LG, R fusiform cx | | |  |  |
|  | | | | | -36 | | | | | | -24 | | | 54 | | | 15772 | | | | <0.001 | | B pre/postCG, L SPL, L IPL, B put,L caudate, L thal, B IC, SMA, L CC, L temp cx, L FC | | |  |  |
|  | | | | | 44 | | | | | | -28 | | | 44 | | | 2792 | | | | <0.001 | | R postCG, R IPL, R SPL | | |  |  |
|  | | | | | -32 | | | | | | -46 | | | -28 | | | 1090 | | | | <0.001 | | L cer IV-V-VI | | |  |  |
|  | | | | | 32 | | | | | | 34 | | | 32 | | | 442 | | | | <0.001 | | R FC | | |  |  |
|  | | | | | -40 | | | | | | 34 | | | 30 | | | 188 | | | | <0.001 | | L FC | | |  |  |
|  | | | | | -36 | | | | | | -44 | | | -50 | | | 129 | | | | <0.001 | | L cer VIII | | |  |  |
| **Deactivation paced** | | | | | 38 | | | | | | -84 | | | 12 | | | 15453 | | | | <0.001 | | precuneous, B V2, B CC | | |  |  |
|  | | | | | -64 | | | | | | -24 | | | -6 | | | 756 | | | | <0.001 | | L temp cx | | |  |  |
|  | | | | | -56 | | | | | | 22 | | | 20 | | | 736 | | | | <0.001 | | L FC | | |  |  |
|  | | | | | 18 | | | | | | -78 | | | -36 | | | 232 | | | | <0.001 | | R cer CRUS I-II | | |  |  |
|  | | | | | -20 | | | | | | -82 | | | -34 | | | 103 | | | | <0.05 | | B cer CRUS I-II | | |  |  |
| **Activation unpaced** | | | | | -36 | | | | | | -24 | | | 54 | | | 24409 | | | | <0.001 | | B pre/postCG, B SPL, SMA, B CC,L IC, L temp cx, B FC, B thal, B put, B IPL, B caudate | | |  |  |
|  | | | | | 20 | | | | | | -48 | | | -20 | | | 6920 | | | | <0.001 | | R cer IV-V-VI-VIII, R LG, R fusiform cx | | |  |  |
|  | | | | | -40 | | | | | | 32 | | | 32 | | | 360 | | | | <0.001 | | L FC | | |  |  |
|  | | | | | -46 | | | | | | -66 | | | 10 | | | 196 | | | | <0.01 | | L temp cx | | |  |  |
|  | | | | | -18 | | | | | | -60 | | | -46 | | | 161 | | | | <0.01 | | L cer VIII-IX | | |  |  |
| **Deactivation unpaced** | | | | | 10 | | | | | | -54 | | | 16 | | | 2588 | | | | <0.001 | | precuneous, B CC | | |  |  |
|  | | | | | 38 | | | | | | -84 | | | 12 | | | 1295 | | | | <0.001 | | precuneous, B V2, B CC | | |  |  |
|  | | | | | -34 | | | | | | -80 | | | 24 | | | 513 | | | | <0.001 | | L IPL | | |  |  |

L : left, R: right, B: bilateral, cx: cortex, CG: central gyrus, SMA: supplementary motor area, cer: cerebellum, PF: prefrontal cortex, IC: insular cortex, SPL: superior parietal cortex, FC: frontal cortex, V1/V2: primary and secondary Visual cortex, OC: occipital cortex, CC: cingulate cortex, LG: lingual gyrus, temp: temporal, put: putamen, IPL: inferior parietal cortex, thal: thalamus, HC: healthy controls; non-PS: non-slowed patients; PS: slowed patients
